# An open repository for single-cell reconstructions of the brain forest

**DOI:** 10.1038/sdata.2018.6

**Published:** 2018-02-27

**Authors:** Masood A. Akram, Sumit Nanda, Patricia Maraver, Rubén Armañanzas, Giorgio A. Ascoli

**Affiliations:** 1 Center for Neural Informatics, Structures & Plasticity, Krasnow Institute for Advanced Study, George Mason University, Fairfax, VA 22030, USA

**Keywords:** Computational neuroscience, Biophysical models

## Abstract

NeuroMorpho.Org was launched in 2006 to provide unhindered access to any and all digital tracings of neuronal morphology that researchers were willing to share freely upon request. Today this database is the largest public inventory of cellular reconstructions in neuroscience with a content of over 80,000 neurons and glia from a representative diversity of animal species, anatomical regions, and experimental methods. Datasets continuously contributed by hundreds of laboratories worldwide are centrally curated, converted into a common non-proprietary format, morphometrically quantified, and annotated with comprehensive metadata. Users download digital reconstructions for a variety of scientific applications including visualization, classification, analysis, and simulations. With more than 1,000 peer-reviewed publications describing data stored in or utilizing data retrieved from NeuroMorpho.Org, this ever-growing repository can already be considered a mature resource for neuroscience.

## Introduction

At the turn of the millennium the field of neuroinformatics was still in its infancy, but many researchers had already embraced computer simulations as an important approach to ground the understanding of the nervous system into quantitative models^1–3^. Pioneering work in anatomy and physiology had long established that the tree-like morphologies of neuronal axons and dendrites play fundamental roles in network connectivity and signal processing. Progress in hardware and software had gradually enabled the practice of digitally reconstructing neuronal morphology three-dimensionally from live microscopic feeds or image stacks^4^. These data were especially useful to carry out comprehensive statistical analyses and to build realistic computational models. However, interactions between experimentalists and theoreticians were the exception rather than the norm, and sporadic data exchange relied on serendipitous peer-to-peer contacts.

In that context, NeuroMorpho.Org was envisioned as an open, public-access, online portal where digital reconstructions of neurons from different animal species, brain regions, cell types, experimental conditions, and acquisition methods could be stored and freely retrieved for re-use in any scientific, educational, or artistic application^5^. The first version of the database was released in 2006 with an initial set of less than 1,000 cells^6^ and was immediately adopted with broad enthusiasm in the neuroscience community^7^. Since then the repository continuously evolved, adding both new data and improved functionality in each of 30 releases over 11 years. Nevertheless, the key operational principles have remained stable: identifying suitable data in peer-reviewed publications, requesting the source files from authors, and making them available to the community after processing into ready-to-reuse form.

Here we describe the current status and future prospects of this mature resource from multiple perspectives, including those of the users, contributors, and developers. We start with an overview of the content and web-based accessibility, then we describe the community usage of these data, and lastly we provide a backstage view of the curation process enabling dense coverage of this data type^8^.

## Results

### Database content

**Figure 1:**
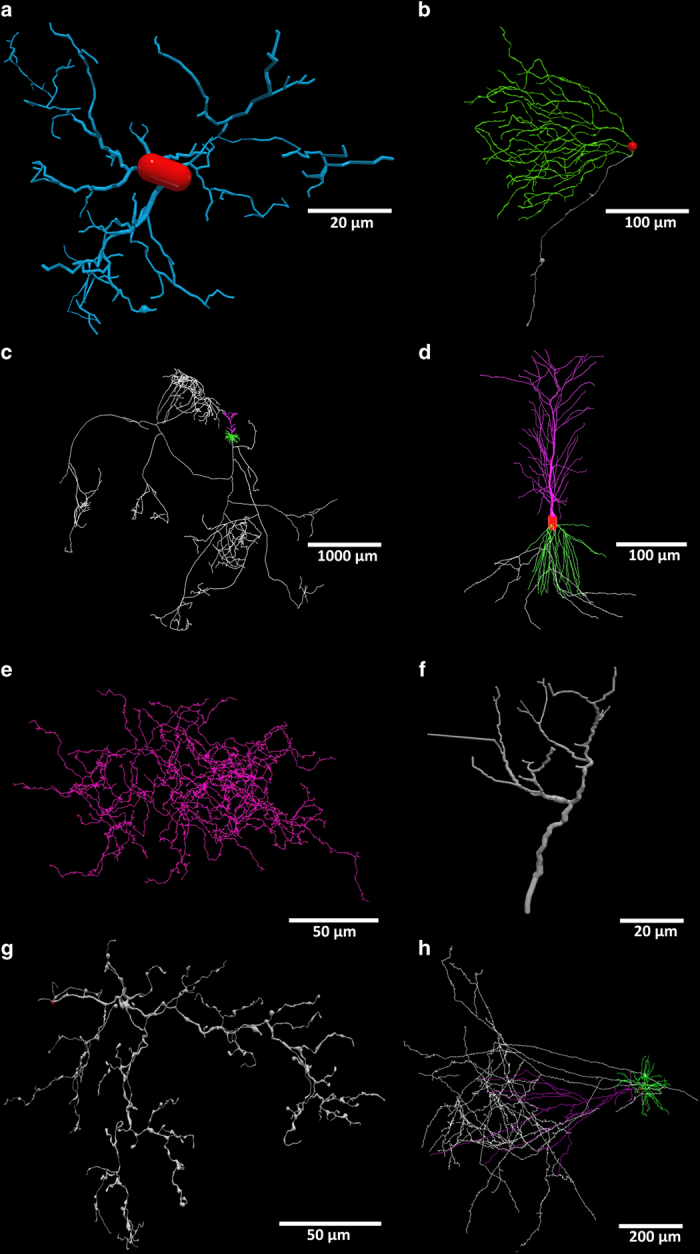
Digital reconstructions of cellular morphologies in NeuroMorpho.Org. (**a**) Microglia cell from mouse spinal cord^52^, with processes in blue and cell body in red (Data Citation 1). (**b**) Ganglion cell from mouse retina^53^, with dendrites in green, axon in silver, and soma in red (Data Citation 2). (**c**) Pyramidal neuron from mouse neocortex^54^, with apical dendrites in magenta, basal dendrites in green, and long projection axon in silver (Data Citation 3). (**d**) Pyramidal neuron from rat hippocampus^55^, with apical dendrites in magenta, basal dendrites in green, and (incomplete) axon in silver (Data Citation 4). (**e**) Interneuron from mouse retina^56^, with unspecified neurites in pink (Data Citation 5). (**f**) Direction sensitive mechanoreceptor from cricket peripheral nervous system, with unspecified neurites in pink^57^ (Data Citation 6). (**g**) Olivocerebellar neuron from rat myelencephalon, with axon in silver^58^ (Data Citation 7). (**h**) Pyramidal neuron from cat neocortex^59^, with apical dendrites in magenta, basal dendrites in green, and long projection axon in silver (Data Citation 8).

Version 7.3 of NeuroMorpho.Org (fall 2017 release) contains 80,012 cells contributed by 416 laboratories and described in 714 peer-reviewed articles. These data come from ~50 anatomical regions of more than 40 animal species ranging from nematodes to humans and notably encompassing both common experimental model (mouse, rat, fruit fly, etc.) and less usual sources (e.g., manatee, leopard, and giraffe). Most pertinently, all major cell types of the nervous system are represented across a number of dimensions (Fig. 1), including sensory receptors, glia, local interneurons and projection neurons releasing excitatory, inhibitory or modulatory neurotransmitters.

For every cell, the database includes the original morphological tracing file as provided by the contributors, the standardized version, and a detailed log of all changes. The basic data format describes the represented trees shape as a set of 3D coordinates (in micrometers), each associated with a tag indicating the type of structure (soma, axon, dendrite, apical, unspecified neurite, glial process or custom-defined), thickness, and the identity of the connected point in the path to the root. In addition to this digital reconstruction file, every cell is displayed with an image, an animation, and a user-interactive in-browser display. For certain users, this latter functionality may require minor JAVA setting adjustments (detailed in the Frequently Asked Questions) due to recent security updates on several operating systems. Moreover, all cells are comprehensively annotated with metadata^9^ regarding the animal subject (species, strain or genotype, sex, age, weight), the cell studied (anatomical region, molecular expression, physiological features), the methodological procedure (experimental condition, tissue sectioning, specimen staining, imaging resolution, tracing system), and linked identifiers (PMID and DOI) of the corresponding referenced publication. Last but not least, a battery of geometric features (length, angles, branch topology, fractal dimension, etc.) is extracted and provided with each individual cell^10^.

### Web functionality

**Figure 2:**
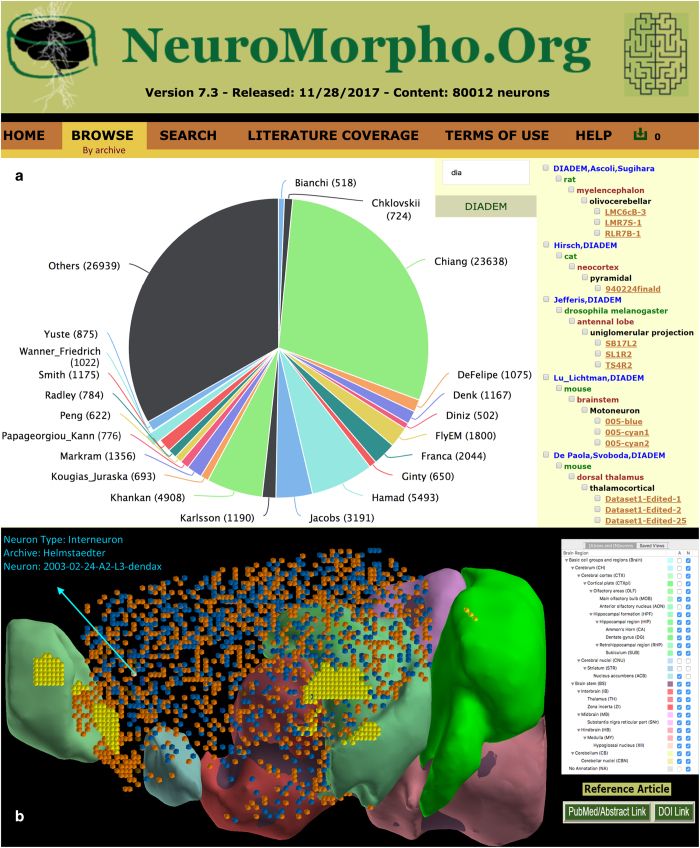
Browse functionality. (**a**) Summary view of selected neurons from the Browse-by-Archive pane, with links to individual entries and options for download. (**b**) NeuronAtlas, a free downloadable application for exploring digital reconstructions of neuronal morphologies from rat and mouse brains.

NeuroMorpho.Org provides a user-friendly graphical interface to access the data through any modern web browser. Visitors can sample a random set of neurons or browse the entire repository by cell type, brain region, animal species, or contributing laboratory, corresponding to the intuitive elements most immediately associated with every study (what, where, which, who). In all cases, data can be selected and downloaded or simply dynamically visualized in rapid sequence with simple cursor movements. Furthermore, the separate Neuron Atlas application, which can be freely downloaded from the Browse menu, repurposes an earlier implementation of the Allen Brain Atlas software to map the spatial distribution of neurons across the major anatomical subdivisions of the rodent brain. The interactive 3D display links to the individual neuron pages and also enables direct visualization of the metadata and the single neuronal morphologies (Fig. 2).

The web front-end also offers multiple search functions. The metadata search option enables one to quickly identify content by conveniently combining filters through logically organized drop-down menus. For example, selecting mouse or drosophila as species populates the strain menu with completely different content. Choosing ‘C57BL/6’ from the former while setting experimental protocol to ‘*in vitro*’ pulls (as of version 7.3, released in November 2017) 4,803 cells, which can be displayed as a summary or downloaded in bulk. The morphometry search is similarly organized allowing users to select the quantitative ranges of geometric parameters of interest. For instance, querying for neurons with at least 100 branches and a maximum path distance from the soma greater than 1 mm retrieves 5,706 cells (v.7.3).

**Figure 3:**
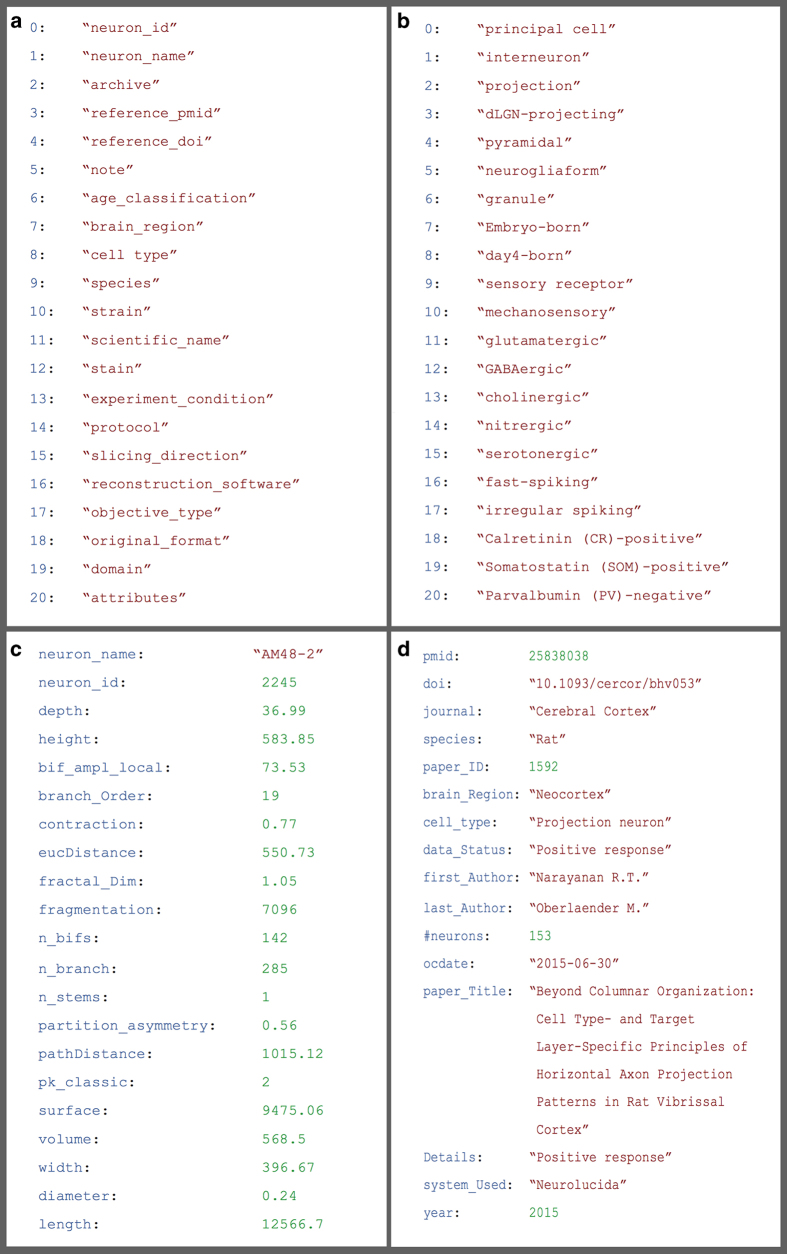
NeuroMorpho.Org’s API gives developers and data miners access to database content and information allowing integration with other neuroinformatics tools. Complete usage documentation and definitions are available at neuromorpho.org/apiReference.html. (**a**) Sample of available JSON objects characterizing experimental methods. (**b**) Selected entries specifying the dimension of cell types. (**c**) Subset of a given cell’s morphometric characteristics defined as attribute-value pairs. (**d**) Bibliographic details for a data set from an individual publication.

A distinct search mechanism adopts a familiar ‘Google-like’ keyword bar with Boolean logic and wildcards. Alternatively, the advanced OntoSearch function leverages logical reasoning by generalization and specification of hierarchically organized knowledge^11^. Moreover, users can query the database content using the Literature Search, which is further elaborated on in the Methods section. The most recently added search functionality makes information machine-accessible through a public Application Programming Interface (API) using JavaScript Object Notation (JSON), a lightweight data-interchange format. The NeuroMorpho.Org API provides large-scale data access through anchors to neuron metadata, morphometric measurements, and literature references, in addition to brain regions, animal species, and more (Fig. 3).

### Usage statistics

Digital reconstructions shared through NeuroMorpho.Org are re-used for various purposes including training and dissemination, implementation of morphologically realistic computational simulations, assessments of potential synaptic contacts, validation of developmental growth models, and comparative statistical analyses across different neuron types, anatomical locations or experimental conditions. The usage of the repository can be quantified in terms of unique website visits, number of downloads, and derived publications. As of December 2017, the web page has been visited above 270,000 times from more than 75,000 unique internet addresses in 166 countries. The number of hits increased from less than 3,000 in the first year to over 15,000 in the June-August 2017 trimester. Notably, two-thirds of this traffic comes from the United States and China.

**Figure 4:**
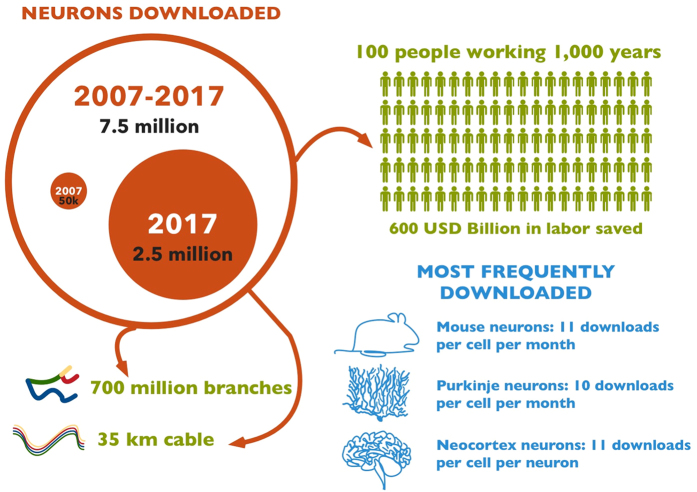
Infographics summary of data download activity from the NeuroMorpho.Org portal.

A total number of 7.5 million neurons were downloaded in standardized format along with 4.5 million ancillary files (original ‘as received’ morphologies and standardization logs) before the 7.3 release. Approximately 50,000 files were downloaded in the first year of the project up to a record 2.5 million in June-August 2017 (Fig. 4). The download volume can also be tallied in number of neurite branches (700 million) or total cable length (35 km). Based on an average of three working days per neuron (one for histology, one for imaging, one for tracing), it would take nearly 100,000 people working for a year to produce the amount of data downloaded in the course of this project. The most popular species and brain region are respectively mouse and neocortex (11 downloads per cell per month). The most frequently downloaded neuron types are Purkinje, newborn granule, and extraverted pyramidal cells (10 downloads per cell per month). Complete statistics are updated online at every release.

The standard way to quantify impact in scientific research is by tracking citations. At least 543 publications cite NeuroMorpho.Org, 353 of which describing results that were directly based on data obtained from this resource. This ‘secondary’ usage substantially adds to the primary service of providing a data repository and standardization process. In this regard, before describing representative examples of re-usage application in the next section, it is worth mentioning that leading publishers, including Springer-Nature, Elsevier, and the Public Library of Science, recommend NeuroMorpho.Org as a trusted database for deposition of digitized morphological reconstructions. This is particularly important given the growing opportunity for ‘data publication’^12^.

### Representative applications

One of the most typical usages of digital reconstructions of neuronal morphology consists of constraining and validating data-driven models of electrophysiological activity or of developmental structural dynamics. For example, pyramidal cells taken from NeuroMorpho.Org are frequently studied in simulations of activity^13^ or structure^14^. Often these studies are extended to obtain novel computational or theoretical conclusions. For instance, the comparative analysis of dendritic complexity across animal taxonomy was recently related to the discriminative capacity of signal integration^15^, and the virtual generation of neuronal trees revealed a balance between the minimization of total wiring and signal conduction time^16^. An open problem increasingly addressed with morphological quantification is the unbiased classification of neuron types^17^. Application of co-clustering techniques on a large set of neocortical cells from many different labs and experimental preparations revealed a clear separation between principal neurons located in different cortical areas and depths^18^. Leveraging modern machine learning methods also yielded methods for content-based retrieval of morphologies, where similar neurons can be efficiently identified from a large collection based on sparse examples^19^.

Earlier examples of secondary discoveries have been extensively reviewed elsewhere^20^. Evidence of broader impact has also emerged beyond basic scientific research. The open availability of digital data is encouraging uptake in divulgation efforts, from undergraduate education^21^ to 3D printable models^22^. By their own nature, many expository or outreach applications are not associated with peer-reviewed references, but are nonetheless beneficial to global societal progress. As representative examples we mention the selection as ‘Site of the Month’ in Neuroscience For Kids (faculty.washington.edu/chudler/neurok.html), the creation of an online teaching module in BrainU (brainu.org), and the use as testing dataset in a past competition of the Chinese Applied Math Olympiads (neuromorpho.org/china_contest.jsp). Similarly, data from NeuroMorpho.Org are used to train commercial expert systems (drvtechnologies.com/aivia5) and to produce multimedia art installations (vimeo.com/191338612). Only a fraction of these outcomes could be predicted at the outset, reinforcing the notion that open data availability may generate genuinely new opportunities for discovery and creativity that would be missed in a more restrictive ‘sharing upon request’ model.

## Discussion

After more than a decade of continuous operation, NeuroMorpho.Org is now considered a stable and mature resource in the neuroscience community. Strikingly, we curators receive as much positive feedback and expressions of gratitude from data contributors as from data users. On the one hand this reflects the value added by the systematic standardization and annotation processes that are detailed in the Methods section. On the other, the above observation strongly refutes the entrenched view of a unidirectional cost-benefit flow from the ‘heroic’ experimental data producers to the ‘parasitic’ computational data modelers^23^. On the contrary, it corroborates the alternative idea of synergistic cooperation even in the absence of direct collaboration, since re-usage demonstrably augments the impact of the original dataset^24^. At the same time, we treasure constructive criticisms from the community, and the vast majority of new and enhanced functionalities were implemented over time to address outside suggestions and requests.

The pace of growth in database content continued to rise substantially over the lifespan of the project. While reconstructions initially accumulated at an average rate of less than 500 per year, most recently the yearly increase of data received passed the figure of 25,000. This rather dramatic acceleration is due to several compounded causes. Most prominently, the field of computational neuroanatomy became increasingly ‘hot’ and the number of publications describing digital reconstruction of neuronal and glial morphology has grown by an order of magnitude over the past 11 years (from ~7 to ~70 articles per month), even faster than the general upward trend in biomedical research in general and neuroscience in particular. Secondly, the progressive automation of the tracing process is boosting the typical dataset size^25^, from less than 20 neurons per study in the first year of operation to over 100 in the most recent one, with a notable single contribution of over 16,000 reconstructions^26^. Third, the attitude towards data sharing is gradually improving, from a hesitant 25% of positive responses in 2006 to a more encouraging 55% in 2017. In order to keep up with the expanding volume of incoming datasets, the information technology and data management infrastructure had to be progressively modernized and improved, as described in the Methods.

The content is also adapting to the parallel evolution of neuroscience research. For instance, glia (astrocytes, oligodendrocytes, microglia, and other types), once considered the ‘dark-matter’ of the nervous system or the ‘forgotten’ brain cells, are now recognized to contribute crucially to fundamental physiological processes, such as neural development^27^, signal processing^28^, and synaptic plasticity^29^. Furthermore, glial cells are involved in major neuropathologies, including Alzheimer’s^30^ and Parkinson’s^31^ diseases, stroke^32^ and epilepsy^33^, as well as traumatic brain^34^ and spinal cord injuries^35^. Specifically, glia morphology represents one of the most useful biomarkers of brain function and dysfunction, as exemplified by enlargement of activated microglia upon rising neuronal death^36^, loss of myelination related to retraction of oligodendrocytes^37^, and altered astrocyte architecture in response to pharmacological treatment^38^ and neurotoxicity^39^. In line with this mounting emphasis and awareness, we started receiving spontaneous requests from researchers to include digital tracings of glia arbor morphology into the repository. We thus appropriately modified the metadata schema, search engine, and ingestion scripts, and release 7.3 now contains 3,069 glial reconstructions from 8 initial datasets.

In the foreseeable future, NeuroMorpho.Org is similarly expected to keep growing both quantitatively (content amount) and qualitatively (content type). Continuous integration with community resources will emphasize neuronal classification both through comprehensive programs such as the National Institutes of Health-fostered Brain Initiative Cell Census Network (BICCN) and domain-specific projects like Hippocampome.Org^40^. This growth is going to necessitate the design and implementation of ergonomic, smart, and robust tools for richer metadata annotation benefiting authors and data curators alike. Moreover, it will be essential to incorporate the anatomical embedding of the reconstructed neurons in a representation of the surrounding tissue. Technically, this only requires the specification of a triad of non-aligned three-dimensional neuronal tracing points relative to a common coordinate framework. This augmented representation is within reach at least for fly, mouse, and human datasets, and would synergistically complement ongoing efforts to capture correlated pre- and post-synaptic circuit connectivity along with the digital morphology^41^. Last but not least, progress is advancing towards the extension of the arbor reconstruction format to accommodate temporal and multi-channel information^42^. These additions are especially important in light of recent experimental breakthrough in live time-lapse super-resolution imaging which could reveal critical biochemical details of neuronal dynamics.

## Methods

**Figure 5:**
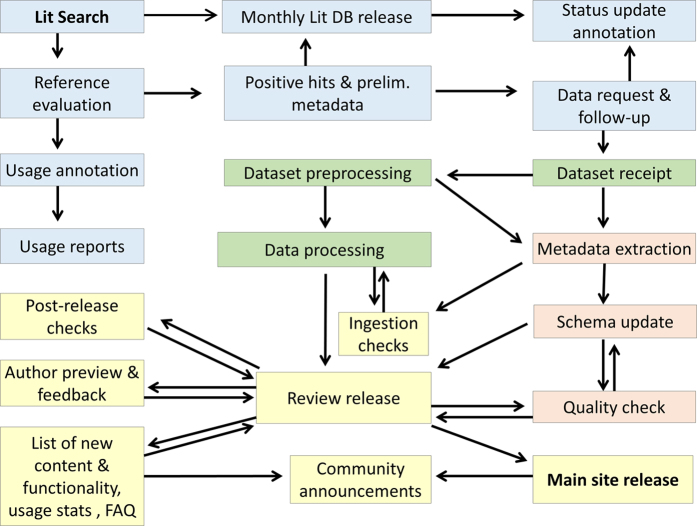
Flowchart of the operational procedures of NeuroMorpho.Org from start (Literature search) to end (Main site release), highlighting literature mining (blue), morphological standardization (green), metadata annotation (pink), and data release (yellow).

This section describes the current operational pipeline of NeuroMorpho.Org. The overall process can be broadly divided into four main task aggregates: literature mining, data processing, metadata annotation, and public release (Fig. 5). The execution of most steps is logically serial for each dataset, though the team organization is largely parallel: while releases are lumped into a few versions per year, new datasets are continuously identified, requested, received, processed, and annotated.

**Figure 6:**
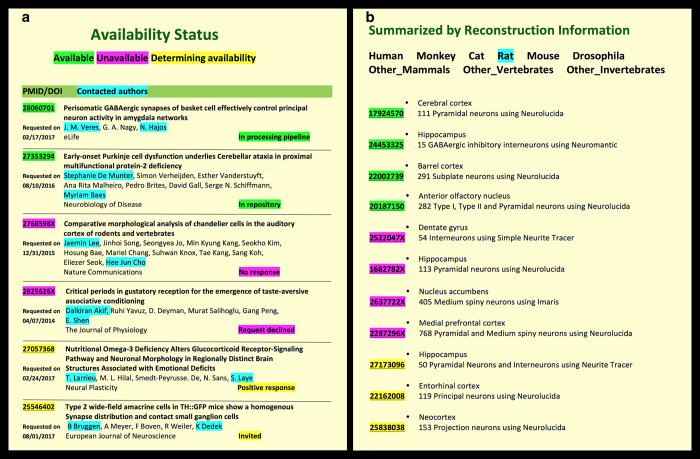
Literature database and search. (**a**) Retrievable information by availability status. To avoid singling out individual authors, the identifiers for declined or unanswered queries are fictional in this illustration; actual unabridged data are publicly posted online in the ‘Literature Coverage’ section of NeuroMorpho.Org. (**b**) Preliminary metadata annotation for all references deemed to describe digital reconstructions of neuronal morphology.

Given NeuroMorpho.Org’s mission of freely distributing digital reconstructions from peer-reviewed publications, it is natural for the data identification process to begin with literature mining^43^. Using a comprehensive battery of keywords in appropriate combinations and several full-text search engines, we collect a set of newly published articles every month which possibly describe tracing datasets. Since the monthly queries were optimized to minimize the number of missed articles, only approximately a third of the shortlisted potential hits actually contain relevant data. Furthermore, users can also directly suggest articles to mine by checking the reference status using the Literature Search function. Team curators carefully evaluate each article and extract preliminary metadata information for every positive instance, including number of reconstructions, brain region, cell type, reconstruction system, bibliographic reference, and corresponding author contact. These minimal details are necessary and sufficient to invite data submission. The outcome of the request (after multiple reminders and follow-ups as needed) determines whether each given dataset is available (received and in processing pipeline) or unavailable (unanswered: ~70%, declined: ~15%, or declared lost: ~15%). A small but increasing proportion of data (5% in 2017) is sent spontaneously by the authors before publication. In addition to new data, the literature mining staff also tracks published usage of data downloaded from the repository. The entirety of the above information is recorded in a public literature database^44^ that is updated monthly (Fig. 6).

If the authors choose to share their traced cells, the next steps are metadata annotation and morphological standardization. The authors are invited to contribute to the metadata annotation; their input is complemented with information extracted from the corresponding publication and team curators formalize the resulting description using control vocabularies and formal ontologies prior to ingestion^11^. New terms are judiciously added as needed to the corresponding schema. Meanwhile, every neuron is assigned a unique machine-readable identifier while also maintaining its original human-assigned name after minor modifications if necessary to avoid occasional duplications or special characters known to interfere with smooth web interactions.

The raw neuronal reconstructions, which can be acquired in more than 20 different tracing systems and file types, are then translated into a common non-proprietary format^45^ and undergo a series of consistency checks such as detection of non-positive diameters, overlapping points, and disconnected branches among many others. A comprehensive description of the complete standardization process is also posted on NeuroMorpho.Org along with the open-source checking software. Inconsistencies are edited using a variety of programs, including CVapp^45^, Neuromantic^46^, neuTube^47^, Vaa3D^48^, TREES Toolbox^49^, and several more as needed^50^. Importantly, all edits are tracked in accompanying log reports.

After completing morphological editing, data processing is finalized by extraction of morphometric parameter and generation of static images^51^ and rotating animations for each neuron. All data, metadata, and ancillary files are then ingested into the previous version of the database to produce a password-protected Review site. This step is essential to enable all contributing authors to preview their data and request changes prior to public release. Furthermore, at this stage we can implement a number of cross-checks, which include: detecting duplicated cells, broken web links, and missing data files; ensuring the accuracy and consistency of new metadata entries and proper integration of enhanced functionalities; and updating usage statistics and frequently asked questions. Upon resolving any ensuing issues if any, the new version is finally openly released and publicly announced through mailing lists and social media.

As with any large-scale scientific database, occasional errors in data or metadata are detected and reported after public release. We try and correct all mistakes immediately if possible, without waiting for the next release. In case of simple typos, the correction is implemented silently. Any change in metadata, in contrast, is disclosed in the following release with a link to detailed documentation (see e.g., last bullet of v6.3 in the ‘What’s New’ page). When correcting the morphological files themselves, a link to the previous version is provided in the Notes of the corresponding neuron page (cf. sixth bullet of v6.1 in the ‘What’s New’ page).

## Additional information

**How to cite this article:** Akram, M. A. *et al.* An open repository for single-cell reconstructions of the brain forest. *Sci. Data* 5:180006 doi: 10.1038/sdata.2018.6 (2018).

**Publisher’s note:** Springer Nature remains neutral with regard to jurisdictional claims in published maps and institutional affiliations.
